# CIÊNCIA, SAÚDE E EPIDEMIAS: OLHARES HISTÓRICOS SOBRE A VARÍOLA

**DOI:** 10.1590/S0104-59702026000100038

**Published:** 2026-11-09

**Authors:** Tânia Salgado Pimenta, Jaqueline Hasan Brizola, Gutiele Gonçalves dos Santos

**Affiliations:** i Pesquisadora, Casa de Oswaldo Cruz, Fundação Oswaldo Cruz, Fiocruz. Rio de Janeiro – RJ – Brasil; ii Pesquisadora em estágio pós-doutoral, Programa de Pós-graduação em História da Ciência e da Saúde, Casa de Oswaldo Cruz, Fundação Oswaldo Cruz, Fiocruz. Rio de Janeiro – RJ – Brasil; iii Doutoranda, Programa de Pós-graduação em História da Ciência e da Saúde, Casa de Oswaldo Cruz, Fundação Oswaldo Cruz, Fiocruz.

O suplemento “Ciência, saúde e epidemias: olhares históricos sobre a varíola” convida à reflexão sobre uma doença que atingiu a humanidade desde a Antiguidade e que marcou profundamente sociedades em diferentes tempos e espaços. A varíola não foi importante apenas na adoção de políticas, práticas médicas e respostas comunitárias ao longo dos séculos, também influenciou de maneira decisiva projetos coloniais, dinâmicas econômicas, fluxos populacionais e formas de administração dos territórios. A doença deixou marcas profundas nas relações sociais, nas estratégias de governo e nas formas de organização do trabalho, especialmente em contextos escravistas e coloniais. No século XX, após sucessivas campanhas internacionais de imunização, esse longo percurso alcançou um marco histórico: em 1980, a Organização Mundial da Saúde declarou oficialmente a erradicação da varíola, que se tornou a primeira doença humana extinta por intervenção sanitária global, um marco sem precedentes na história da saúde pública (Hochman, 2011).

A produção historiográfica sobre medicina tem se consolidado, ao longo de décadas, com estudos importantes sobre as epidemias de varíola, o desenvolvimento da vacina, a sua circulação e os movimentos antivacina, em especial, no Norte Global (Porter, Roy, 1988; Bennett, 2020). Na historiografia brasileira, a varíola se destaca em obras clássicas desenvolvidas com base em diferentes abordagens sobre a Revolta da Vacina, que ocorreu no Rio de Janeiro em 1904 (Chalhoub, 1996; Sevcenko, 1984). Nos últimos anos, sobretudo na área da história das ciências e da saúde, pesquisas vêm colaborando para o entendimento mais amplo e diverso das experiências sociais com essa doença, dialogando com a historiografia internacional sobre o tema em diversos contextos (Agostoni, 2015; Oliveira, 2013; Soumonni, 2012). Nesse sentido, a partir de uma tradição de estudos já consolidada, apresentamos trabalhos com variadas perspectivas e contextos geográficos, que examinam as múltiplas dimensões da varíola e da vacina, contribuindo para compreendermos melhor e de forma mais aprofundada as questões sociais, políticas e médicas em torno da varíola fundamentadas em historiografia não eurocentrada. As reflexões propostas abordam as formas de controle e resistência social diante da doença, os agentes envolvidos na sua prevenção e as redes de circulação de saberes, pessoas e tecnologias no mundo atlântico. Ao iluminar experiências diversas, os artigos demonstram como a varíola foi, simultaneamente, um desafio epidemiológico e um fenômeno social, político e cultural. Três grandes eixos são articulados neste suplemento: as formas como as populações e as autoridades lidaram com a varíola e enfrentaram essa doença; os agentes e redes envolvidos na circulação de técnicas, saberes e materiais; e as conexões atlânticas que vincularam práticas de vacinação e estratégias em diferentes regiões do mundo.

Este suplemento foi motivado pelos resultados do primeiro Seminário Internacional “Ciência, saúde e epidemias: um olhar histórico sobre a varíola”, realizado nos dias 14 e 15 de maio de 2024 na Casa de Oswaldo Cruz (COC), unidade da Fiocruz localizada no Rio de Janeiro. O evento, promovido em formato virtual e transmitido pelo canal da COC no YouTube, reuniu especialistas de diversas instituições públicas do Brasil e de outros países, como Argentina, Chile, México e Espanha (Ciência..., 14 maio 2024). O encontro, organizado por Tânia Pimenta, Jaqueline Brizola e Gutiele dos Santos, com apoio do Departamento de Pesquisa e do Programa de Pós-graduação em História das Ciências e da Saúde da COC, teve o objetivo de discutir as manifestações da varíola desde as grandes epidemias da época moderna e contemporânea até a emergência da vacina, sua difusão e a recepção do novo método em partes dos continentes europeu e americano.

Ao tratar do tema em uma região da Argentina em seu artigo “Instrumentos y agentes de control: la viruela en el Río de la Plata a comienzos del siglo XIX”, María Silvia Di Liscia analisa as ações dos agentes responsáveis pela vacina e oferece, também, um panorama das tensões sociais envolvendo indígenas, vacinadores e administradores do Estado. Ricardo Wan Moguel apresenta um caminho semelhante em “Cien años de vacunación en Yucatán, México, 1802-1902”, em que problematiza os acontecimentos relacionados à vacina jenneriana nos cem anos que se sucedem à chegada da expedição de Francisco Xavier de Balmis à península, em 1802, até as campanhas de vacinação experimentadas no início do século XX. Paula Caffarena, em “Respuestas y resistencias en la lucha contra la viruela: Chile, siglos XVIII-XX”, por sua vez, examina longamente o fortalecimento institucional e a obrigatoriedade da vacinação, identificando uma resistência passiva à vacinação.

O artigo de José Luís Duro Torrijos, “Recursos tecnológicos en el inicio de la difusión de la vacuna contra la viruela en Europa y América”, mostra como inovações técnicas e redes institucionais foram fundamentais para viabilizar a circulação da vacina antivariólica, sobretudo a partir de recursos logísticos e tecnológicos empregados na conservação e no transporte do fluido vacinal no princípio do século XIX.

Jaqueline Hasan Brizola e Gutiele Gonçalves dos Santos focalizam a chegada e a disseminação da vacina antivariólica no contexto do Império português em “Entre braços e lâminas: interesses escravistas na distribuição da vacina antivariólica no Brasil e em Angola, 1804-1810”. O estudo mostra que as conexões entre territórios coloniais eram mais amplas, atentando a relação entre Bahia e Angola quanto a ações imperiais voltadas para o combate às epidemias de varíola. Diante da disseminação da vacina, o artigo de Fillipe Portugal e Maria Rachel Fróes, “‘A prática da inoculação da vacina e as suas funestas consequências’: uma obra contra a vacina antivariólica no Império português no início do XIX”, analisa os debates sobre vacina e medicina na primeira metade do século XIX, aprofundando o estudo sobre perspectivas médicas, sociais e científicas contrárias à vacina.

No artigo “Sob as marcas de bexigas: varíola e africanos na Corte imperial na primeira metade do século XIX”, Tânia Pimenta e Flavio Gomes identificam o impacto da varíola sobre africanos e seus descendentes no Rio de Janeiro em inícios do Oitocentos, apontando que as sequelas, em grande parte em forma de marcas na pele em quem sobrevivia, constituíam um sinal da escravidão alardeado nos anúncios de fuga, roubo ou venda.

Benedito Barbosa analisa, no artigo “Os povos indígenas à sombra das ‘perniciosíssimas’ bexigas na Amazônia portuguesa, 1757-1819”, os impactos devastadores das epidemias de bexigas entre as populações indígenas da Amazônia portuguesa na segunda metade do século XVIII. O autor demonstra como os surtos da doença não apenas dizimaram indígenas, mas também desestruturaram profundamente a dinâmica demográfica e o crescimento econômico da região.

O suplemento traz também duas entrevistas com importantes estudiosas da história da varíola e da vacina. Na Espanha, Susana Ramirez Martín, professora da Universidade Complutense de Madrid, tornou-se referência para a compreensão dos acontecimentos relacionados à difusão da vacina jenneriana na América em princípios do século XIX com seus estudos sobre a Expedição Filantrópica da Vacina, considerada a primeira tentativa de vacinação em massa da história. No Brasil, Tania Maria Dias Fernandes, pesquisadora aposentada da COC, desenvolveu trabalho pioneiro sobre a história da vacina antivariólica e a erradicação da varíola, incontornável para quem se dedica ao tema.

Os artigos e as entrevistas publicados oferecem, em conjunto, um panorama amplo e multifacetado sobre a história da varíola, dos conhecimentos produzidos em torno dessa doença e das medidas contra as epidemias que contribuem para a historiografia e para a discussão pública com a expectativa de que outros estudos sejam incentivados. Para tanto, foram fundamentais as contribuições dos autores e pareceristas, a quem agradecemos.

Ao organizar esse suplemento da revista *História, Ciências, Saúde – Manguinhos*, consideramos relevante revisitar a história da varíola em um momento em que discussões sobre saúde global, vacina e circulação de agentes patogênicos voltam ao centro do debate público, em que são identificados movimentos anticiência e antivacina difundidos em diversas regiões do mundo. Essa reflexão histórica se torna, portanto, ainda mais necessária e provocadora.

## References

[B1] AGOSTONI Claudia (2015). Control, contención y educación higiénica en las campañas de vacunación contra la viruela en México durante la década de 1940. História, Ciências, Saúde - Manguinhos.

[B2] BENNETT Michael (2020). War against smallpox: Edward Jenner and the global spread of vaccination.

[B3] CHALHOUB Sidney (1996). Cidade febril: cortiços e epidemias na Corte imperial.

[B4] (2024). CIÊNCIA, saúde e epidemias: um olhar histórico sobre a varíola.

[B5] HOCHMAN Gilberto (2011). Vacinação, varíola e uma cultura da imunização no Brasil. Ciência & Saúde Coletiva.

[B6] OLIVEIRA Eliézer Cardoso de (2013). A epidemia de varíola e o medo da vacina em Goiás. História, Ciências, Saúde - Manguinhos.

[B7] PORTER Dorothy, PORTER Roy (1988). The politics of prevention: anti-vaccinationism and public health in the nineteenth-century England. Medical History.

[B8] SEVCENKO Nicolau (1984). A Revolta da Vacina: mentes insanas em corpos rebeldes.

[B9] SOUMONNI Elisée (2012). Disease, religion and medicine: smallpox in nineteenth century Benin. História, Ciências, Saúde - Manguinhos.

