# Supplementary material for: CIÊNCIA, SAÚDE E EPIDEMIAS: OLHARES HISTÓRICOS SOBRE A VARÍOLA
Source: Hist Cienc Saude Manguinhos. 2026 Nov 9;33(Suppl 1):e2026038. [Article in Portuguese] doi: 10.1590/S0104-59702026000100038 (PMC13750634; doi:10.1590/S0104-59702026000100038)
Supplement: Translation

# Translation: SCIENCE, HEALTH AND EPIDEMICS: HISTORICAL PERSPECTIVES ON SMALLPOX

**DOI:** 10.1590/S0104-59702026000100038en

**Authors:** Tânia Salgado Pimenta, Jaqueline Hasan Brizola, Gutiele Gonçalves dos Santos

**Affiliations:** i Researcher, Casa de Oswaldo Cruz, Fundação Oswaldo Cruz, Fiocruz. Rio de Janeiro – RJ – Brazil; ii Post-doctoral researcher, Graduate Program in the History of Science and Health, Casa de Oswaldo Cruz, Fundação Oswaldo Cruz, Fiocruz. Rio de Janeiro – RJ – Brazil; iii Doctoral student, Graduate Program in the History of Science and Health, Casa de Oswaldo Cruz, Fundação Oswaldo Cruz, Fiocruz.

The supplement entitled “Science, health and epidemics: historical perspectives on smallpox” invites us to reflect on a disease that has affected humanity since ancient times and profoundly affected societies across different times and places. Smallpox was important not only in the adoption of policies, medical practices and community responses over the centuries, but also had a decisive influence on colonial endeavors, economic dynamics, population flows and ways of administering territories. This disease deeply affected social relations, government strategies, and ways of organizing work, especially in contexts involving slavery and colonialism. During the twentieth century, after successive international immunization campaigns, this long journey reached a historic milestone: in 1980 the World Health Organization officially declared the eradication of smallpox, which was the first human disease to be eliminated as the result of global health interventions, an unprecedented achievement in the history of public health (Hochman, 2011).

Historiographic work on medicine has been consolidated over the decades with important studies on smallpox epidemics, development of the vaccine, circulation, and anti-vaccine movements, particularly in the Global North (Porter, Roy, 1988; Bennett, 2020). In the Brazilian historiography, smallpox stands out in classic works based on different approaches to the Vaccine Revolt that took place in Rio de Janeiro in 1904 (Chalhoub, 1996; Sevcenko, 1984). In recent years, especially in the area of the history of science and health, research has contributed to a broader and more diverse understanding of social experiences involving this disease, creating a dialog with international historiography on this subject in various contexts (Agostoni, 2015; Oliveira, 2013; Soumonni, 2012). In this sense, based on an established tradition of research, we present studies from a variety of perspectives and geographical contexts that examine the multiple dimensions of smallpox and its vaccine, contributing to a better and more in-depth understanding of the social, political, and medical issues surrounding smallpox from non-Eurocentric historiography. The reflections collected here address forms of social control and resistance to the disease, agents involved in prevention, and the networks through which knowledge, people and technologies circulated in the Atlantic world. By shedding light on diverse experiences, the articles show how smallpox was simultaneously an epidemiological challenge and a social, political and cultural phenomenon. Three main themes interconnect in this supplement: the ways in which populations and authorities dealt with smallpox and confronted the disease; the agents and networks involved in the circulation of techniques, knowledge and materials; and the Atlantic connections that linked vaccination practices and strategies in different regions of the world.

This supplement was motivated by the results of the first International Seminar entitled “Ciência, saúde e epidemias: um olhar histórico sobre a varíola” [Science, health and epidemics: a historical look at smallpox], which was held on May 14 and 15, 2024 at the Casa de Oswaldo Cruz (COC), a Fiocruz unit located in Rio de Janeiro. The event, which also involved virtual participation and was broadcast on COC’s YouTube channel, brought together experts from various public institutions in Brazil and other countries such as Argentina, Chile, Mexico and Spain (Ciência..., 14 maio 2024). The aim of the meeting, organized by Tânia Pimenta, Jaqueline Brizola and Gutiele dos Santos with support from the Research Department and the Graduate Program in the History of Science and Health at COC, was to discuss the manifestations of smallpox, ranging from the great epidemics of modern and contemporary times to the emergence of the vaccine, its diffusion, and how the new method was received in areas of Europe and the Americas.

In her article “Instrumentos y agentes de control: la viruela en el Río de la Plata a comienzos del siglo XIX,” [Instruments and agents of control: smallpox in the Río de la Plata at the beginning of the nineteenth century] which addressed this topic in one region of Argentina, María Silvia Di Liscia analyzes the actions of the agents responsible for the vaccine and also offers an overview of the social tensions involving indigenous people, vaccinators and government administrators. Ricardo Wan Moguel takes a similar path in “Cien años de vacunación en Yucatán, México, 1802-1902,” [One hundred years of vaccination in Yucatán, México, 1802-1902] where he examines events related to the Jennerian vaccine over the hundred years following the arrival of Francisco Xavier de Balmis’ expedition on the peninsula in 1802, up until the experimental vaccination campaigns at the beginning of the twentieth century. Paula Caffarena, in “Respuestas y resistencias en la lucha contra la viruela: Chile, siglos XVIII-XX,” [Responses and resistance in the fight against smallpox, Chile, eighteenth to twentieth centuries] undertakes a long-term analysis to investigate institutional strengthening and the obligatory nature of vaccination, identifying passive resistance.

The article by José Luís Duro Torrijos, “Recursos tecnológicos en el inicio de la difusión de la vacuna contra la viruela en Europa y América” [Technological resources during the early dissemination of the smallpox vaccine in Europe and the Americas], shows how technical innovations and institutional networks were fundamental in enabling circulation of the smallpox vaccine, especially by means of the logistical and technological resources used in to preserve and transport the vaccine fluid in the early nineteenth century.

Jaqueline Hasan Brizola and Gutiele Gonçalves dos Santos focus their analysis on the arrival and dissemination of the smallpox vaccine within in the context of the Portuguese Empire in “Entre braços e lâminas: interesses escravistas na distribuição da vacina antivariólica no Brasil e em Angola, 1804-1810” [In arms and on slides: slavery-related interests in the dissemination of smallpox vaccine in Brazil and Angola, 1804-1810]. This study shows that the connections between colonial territories were broader, examining the relationship between Bahia and Angola in terms of imperial actions to combat smallpox epidemics. Considering dissemination of the vaccine, the article by Fillipe Portugal and Maria Rachel Fróes entitled “‘A prática da inoculação da vacina e as suas funestas consequências’: uma obra contra a vacina antivariólica no Império português no início do XIX” [“The practice of vaccine inoculation and its dire consequences”: a work against the anti-smallpox vaccine in the Portuguese Empire in the early-nineteenth century] analyzes debates on vaccines and medicine in the first half of the nineteenth century and delves into medical, social and scientific perspectives opposing the vaccine.

In the article “Sob as marcas de bexigas: varíola e africanos na Corte imperial na primeira metade do século XIX” [Under the marks of pustules: smallpox and Africans in the Imperial Court in the first half of the nineteenth century], Tânia Pimenta and Flavio Gomes identify the impact of smallpox on Africans and their descendants in Rio de Janeiro in the early nineteenth century, pointing out that the signs of the disease (generally marks on the skin of those who survived) served as a sign of slavery that was flaunted in notices of escape, theft or sale.

And in the article “Os povos indígenas à sombra das ‘perniciosíssimas’ bexigas na Amazônia portuguesa, 1757-1819” [The indigenous peoples under the “highly pernicious” pustules in the Portuguese Amazon, 1757-1819], Benedito Barbosa examines the devastating effects of smallpox epidemics on the Indigenous populations of Portuguese Amazonia in the second half of the eighteenth century. The author demonstrates how outbreaks of the disease not only decimated Indigenous communities but also profoundly disrupted the region’s demographic dynamics and economic growth.

The supplement also features two interviews with important scholars on the history of smallpox and the vaccine. In Spain, Susana Ramirez Martín, a professor at the Complutense University of Madrid, has become a reference in understanding the events related to the spread of the Jennerian vaccine across the Americas at the beginning of the nineteenth century with her studies on the Philanthropic Vaccine Expedition, considered history’s first attempt at mass vaccination. In Brazil, retired COC researcher Tania Maria Dias Fernandes carried out pioneering work on the history of the smallpox vaccine and eradication of the disease.

Published together, these articles and interviews offer a broad and multifaceted overview of the history of smallpox, the knowledge produced about this disease, and the measures taken against epidemics, which contribute to the historiography and public discussion and are expected to spark further studies. The contributions of the authors and referees were essential in this process, and we wish to express our appreciation.

In organizing this supplement to the journal *História, Ciências, Saúde – Manguinhos*, we believe revisiting the history of smallpox is relevant at a time when discussions about global health, vaccines, and the circulation of pathogens are once again at the center of public debate, and when anti-science and anti-vaccine movements are widespread in various regions of the world. Against this backdrop, this historical reflection becomes even more necessary and provocative.
